# Transgenerational effects on body size and survival in Brook charr (*Salvelinus fontinalis*)

**DOI:** 10.1111/eva.13553

**Published:** 2023-05-02

**Authors:** Carolyne Houle, Philippine Gossieaux, Louis Bernatchez, Céline Audet, Dany Garant

**Affiliations:** ^1^ Département de Biologie Université de Sherbrooke Sherbrooke Québec Canada; ^2^ Institut de Biologie Intégrative et des Systèmes (IBIS) Université Laval Québec City Québec Canada; ^3^ Institut des Sciences de la Mer de Rimouski (ISMER) Université du Québec à Rimouski (UQAR) Rimouski Québec Canada

**Keywords:** morphological traits, parental effects, salmonids, survival, transgenerational effects

## Abstract

Higher temperatures are now observed in several ecosystems and act as new selective agents that shape traits and fitness of individuals. Transgenerational effects may be important in modulating adaptation of future generations and buffering negative impacts of temperature changes. The potential for these effects may be important in freshwater fish species, as temperature is a key abiotic component of their environment. Yet, still, relatively few studies have assessed the presence and importance of transgenerational effects under natural conditions. The purpose of this study was to test how parental thermal conditions influenced offspring growth and survival following stocking in Brook charr (*Salvelinus fontinalis*). To do so, part of the breeders were exposed to a “cold” treatment while others were exposed to a “warm” treatment during the final steps of gonad maturation (constant 2°C difference between treatments along the seasonal temperature decrease). The impact on offspring of a selection treatment targeting production traits of interest (absence of sexual maturation at 1+, combined with increased growth) in breeders was also evaluated. After 7–8 months of growth in captivity, offspring were stocked in natural lakes. Their growth and survival were assessed about a year later. Offspring from “cold” breeders showed lower survival than those from “warm” breeders and the selection treatment had no effect on survival. However, the selection treatment was linked to lower Fulton's condition index, which, in turn, was positively correlated to survival in lakes. This study highlights the importance of working in ecological/industrial context to fully assess the different impacts of transgenerational effects on traits and survival. Our results also have important implications for stocking practices used to support the sport fishing industry.

## 1 INTRODUCTION

Climate changes already affect biomes around the world and their impact should further increase in forthcoming decades (IPCC, 2021). Environmental conditions have changed drastically in most ecosystems due to increasing temperatures, which are expected to keep rising (IPCC, 2021; Schleussner et al., 2016). These elevated temperature conditions often act as new selective agents that shape phenotypic and genetic variation within populations and modulate the effect of phenotypes on the fitness of individuals (Crozier & Hutchings, 2014; Gienapp et al., 2014). It is thus crucial to determine how these temperature changes ultimately affect wild populations and in particular, if natural populations will be able to adapt to changing conditions (Chevin et al., 2010; Fox et al., 2019).

One way organisms can adapt to changing environments is via transgenerational effects, where conditions experienced in the parental generation may influence offspring performance. Transgenerational effects may thus be important in buffering negative impacts on, and modulating the adaptation of, future generations to temperature changes (Anastasiadi et al., 2021; Evans et al., 2014; Guillaume et al., 2016; Salinas & Munch, 2012). For example, parents exposed to different temperature conditions may incur long‐lasting effects on their offspring's traits and/or fitness. Adaptive transgenerational effects should be favoured under changing environmental conditions when parents can anticipate their offspring's environments (Donelson et al., 2018; Herman & Sultan, 2011). Yet, the importance of transgenerational effects in the wild is still debated, especially in cases when the environment is variable among generations (Sánchez‐Tójar et al., 2020; Uller et al., 2013; Yin et al., 2019). Moreover, paternal effects have been much less studied than maternal effects, despite their increasingly recognized importance (McAdam et al., 2014; Penney et al., 2022; Rutkowska et al., 2020). Transgenerational studies assessing the effect of temperature variation during maturation/reproduction of both males and females on their subsequent juvenile morphology and survival until maturation/reproduction in the wild are thus still needed (Donelson et al., 2018).

Temperature is a key abiotic component of freshwater ecosystems that affects phenological and physiological processes at different levels of biological organisation and in various aquatic taxa (Alfonso et al., 2021; Ficke et al., 2007; Knouft & Ficklin, 2017; Lynch et al., 2016; Whitney et al., 2016; Winder & Schindler, 2004; Woodward et al., 2010). In fishes, for instance, the temperature is critical for the onset of sexual maturation and reproduction as well as for the development and survival of early life stages (reviewed in Pankhurst & King, 2010; Pankhurst et al., 2011). Transgenerational effects related to temperature may thus play an important role in modulating the impact of temperature fluctuations on the traits and survival of offspring. For example, exposure to elevated temperature during gametogenesis often results in reduced maternal investment or gamete viability (Pankhurst & King, 2010). Warren et al. (2012) showed that an increase of 1°C of the summer mean of maximum daily air temperature could be enough to delay Brook charr (*Salvelinus fontinalis*) spawning in the fall. Also, Spinks et al. (2022) showed that parental exposure to warm conditions (+1.5°C), in a coral reef fish (*Acanthochromis polyacanthus*), resulted in decreased weight and condition of their offspring. Yet, a recent study in controlled lab conditions in lake trout (*Salvelinus namaycush*) found little evidence of transgenerational effects for adults acclimated to either cold or warm temperatures on their offspring's thermal performance later on (see Penney et al., 2021). These mixed results thus highlight the need for more studies of transgenerational effects in fishes.

Brook charr is a widely distributed salmonid in rivers and lakes of eastern North America. In Québec, in particular, it is the most important species for recreational angling activities (MFFP, 2019). To keep up with the demand of angling activities, Brook charr has been domesticated and repeatedly stocked in North American lakes for many years (Lehnert et al., 2020; White et al., 2018). However, high post‐stocking mortality and decreased body condition following stocking have been reported in this species (Ersbak & Haase, 1983; Simpkins & Hubert, 2011). Thermal tolerance is shown to vary among strains of Brook charr, with individuals from northern latitude having more limited thermal tolerance to warm conditions (Stitt et al., 2014). A recent study in controlled conditions, using Brook charr families produced by parents acclimated to cold or warm temperatures, showed limited response to warmer temperatures, but revealed that sires appeared to have greater contributions to transgenerational effects than dams (Penney et al., 2022). Another study found 188 differentially methylated regions in progenies issued from breeders exposed to different temperature conditions during the final steps of gonad maturation (Venney et al., 2022), which could possibly translate into different phenotypes and fitness for those fish.

The objective of this study was to test how parental effects in Brook charr may affect traits of importance for stocking success, i.e. survival and growth. To do so, we exposed breeders to one of two different temperature regimes applied during the final gonad maturation steps and maintained a constant 2°C difference among treatments to reflect what is expected by 2100 in Canada in terms of freshwater warming, under a low emission scenario (RCP 2.6; Zhang et al., 2019).

Fish stocked in natural environments are issued from aquaculture production where selection of traits of interest is a common practice. Yet, relatively little is known about how artificially selected individuals perform compared to unselected ones once they are released in the wild and how such selection shapes parental effects (Bastien et al., 2011; Biro et al., 2004). Here we used two lines of the Laval strain (1‐ a line selected for the absence of sexual maturity at age 1+ and growth, which is called selected hereafter and 2‐ a line without selection, which is called unselected hereafter). We then assessed how transgenerational effects may have impacted the survival of offspring 1 year after being stocked in natural lakes and if the selection process used during offspring production interfered with these transgenerational effects.

## 2 METHODS

### 2.1 Breeding design

All treatments and manipulations on adults were conducted at the *Station piscicole de l'Institut des sciences de la mer de Rimouski* (ISMER), Rimouski, Québec. Experiments were conducted on adult Brook charr of the Laval strain issued from the production programme held at ISMER. This strain comes from an anadromous population that originates from the Laval River, near Forestville, Québec (Bastien et al., 2011). We used two lines of adults maintained in our facilities: (1) the ‘Selected’ line, issued from a selection programme aiming to eliminate sexual maturation and improve growth at age 1+, and (2) the ‘Unselected’ line, on which no selection process was applied (even though a domestication process certainly occurred). Crosses were made randomly in the unselected line while avoiding crossings between brothers and sisters. Breeders from the 5th and 6th generation of production (both lines) were used for this study.

**FIGURE 1:**
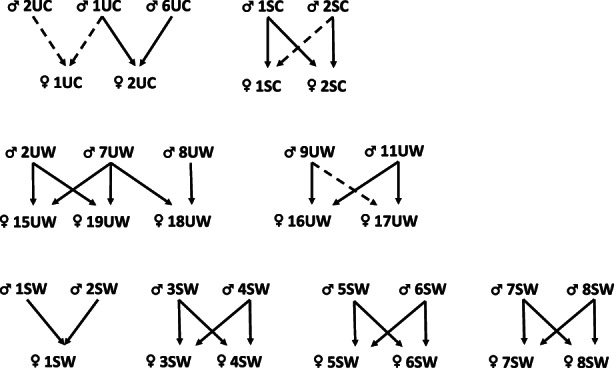
Diagram of crosses made in fall 2018. Symbols for males (♂) and females (♀) are followed by individual number and letters identifying lineage (S, selected; U, unselected) and treatments (C, cold; W, warm) respectively. Solid arrows indicate families that survived until exogenous feeding stage.

The Laval strain is an anadromous strain, 2+ and older individuals are thus reared in saltwater from June to September and then returned to freshwater. Aerated flow‐through tanks are filled either from salt water pumped from the St. Lawrence estuary or from dechlorinated city water (surface water source). Temperature, salinity and oxygen are monitored daily. Temperature is monitored with thermometers placed inside the rearing tanks, salinity is adjusted using a salinity refractometer (Aquafauna Bio‐Marine), and adequate oxygenation is provided with aeration system present in the flow‐through rearing tanks. In September 2018, breeders from both selection regimes were separated into two groups in order to expose them, during the late gonad maturation period, to two different thermal regimes: a “Cold” group with temperature gradually decreasing from 11.5°C in September to 3°C in December, and a “Warm” group with temperature gradually decreasing from 13.5°C in September to 5°C in December (Figure S1). When female ovulation occurred (mid‐November to mid‐December 2018), eggs and sperm were extracted manually and crosses, following a specific pattern, were made within each group. Whenever possible, each adult was crossed with two individuals of the opposite sex from the same treatment. This resulted in 32 families being produced, of which 28 families issued from 16 females and 18 males survived until exogenous feeding (Figure 1). All individuals from family ♂8SW × ♀8SW died at the exogenous feeding stage, leaving 27 families at stocking stage (Table S1): two unselected‐cold families; three selected‐cold families; nine unselected‐warm families; 13 selected‐warm families. Thus, as shown in Figure 1, fewer families were obtained for the cold than for the warm treatment. Eggs, fry and juveniles were maintained in flow‐through dechlorinated freshwater systems and all individuals were exposed to the same thermal regime. Eggs and fry were raised keeping each family separated using flow‐through systems (see Bastien et al., 2011). The thermal regime for egg incubation varied from a maximum of 10.5°C depending on the ovulation timing to a minimum of 4°C (natural seasonal water temperature decrease). Heaters were then used to maintain the water temperature at 4°C until hatching. Eggs were incubated in the dark. At hatching (from the beginning of February until the end of March), the temperature was gradually increased at a rate of 1°C per week from 4°C to 8°C at first feeding. Photoperiod was adjusted to 12 L/12D. In June 2019, when temperature naturally reached 8°C, heaters were stopped and juveniles were then reared under natural temperature and photoperiod conditions all year long. Fry were maintained in separate rearing units until late June to ensure they were large enough for fin clipping. At this stage, 500 juveniles per family (chosen randomly in small numbers at a time, and from different regions of the rearing unit each time) were marked according to the family group by different combinations of fin clippings (adipose and pelvic: eight combinations possible including the absence of fin clipping) and transferred to 250 L flow‐through dechlorinated rearing tanks, using random combinations of 5 or 6 families per fish tank. At the end of August 2019, fish number per family was reduced to 250 (randomly, as described above), marks were verified and refreshed if necessary, and juveniles were transferred to 500 L tanks. Fish were maintained at a density that never exceeded 30 kg/m^3^.

### 2.2 First year in a laboratory environment

In September 2019, juvenile mass (weight, in g) was measured for approx. 30 individuals per family (total *N* = 803; N for Cold and Unselected treatment = 60; N for Cold and Selected treatment = 90; N for Warm and Unselected treatment = 270; N for Warm and Selected treatment = 383) and pictures were taken to assess body length (FL, in cm) by placing landmarks at the most anterior and posterior parts of the body in tpsDig v.2.31 (Rohlf, 2005) and by measuring the distance between those landmarks via CoordGen8's TMorphGen8 tool (Sheets, 2014). We used these measurements to calculate Fulton's body condition index (K) for each individual as: *K* = 100 × weight/FL^3^ (Fulton, 1904).

### 2.3 Second year in a laboratory environment

Non‐stocked individuals from all families were maintained in the conditions described above. However, numbers and mix of families varied over time, as randomly captured individuals were used for other concurrent experiments performed using these groups of fish (ex. determination of CT_max_ and evaluation of thermal tolerance). As fish were maintained under our standard rearing conditions [feeding 7 days a week from September to December; feeding 2 days a week from December to April; feeding 7 days a week starting in April; feed rations calculated based on tank loads (maximum 30 kg m^−3^) with care taken to never be below satiation], we used them to compare their growth with growth measured in recaptured stocked fish. In June 2020, the same measurements (body mass, length and Fulton's condition index) were taken on a total of 1368 individuals issued from different families (N for Cold and Unselected treatment = 100; N for Cold and Selected treatment = 149; N for Warm and Unselected treatment = 443; N for Warm and Selected treatment = 676).

### 2.4 Stocking and sampling of second‐year fish in lakes

A group of fish representing all families and treatments was stocked (total *N* = 3611, mean family^−1^ = 133) in two lakes (Lac de l'Allemand and Lac de l'Arche) of approx. 5–6 ha each located in the Pourvoirie des Bouleaux Blancs, Forestville, Québec (48°43′55″ N, 69°14′02″ W), in October 2019 (after 7–8 months of growth in captivity). In September 2020, after 1 year of growth in natural environment, we captured fish over 6 days (total number caught, *N* = 184) using a combination of gill nets (*N* = 126) and fishing rods (*N* = 58). Each sampled fish was weighted, and a picture was taken to measure its body length and to calculate Fulton's body condition index (K). The protocol and procedures employed were ethically reviewed and approved by University Animal Care Committees operating under the Canadian Council on Animal Care guidelines (protocol #2020–2689, U. Sherbrooke).

### 2.5 Genetic analyses and parentage assignment

DNA was extracted from adults used for crosses in 2018 and from individuals caught in 2020, following the protocols described in Gossieaux et al. (2018). All samples were then amplified at 12 microsatellite loci and visualised on an AB3500 automated DNA sequencer (Applied Biosystems) using GeneScan 600 LIZ size standard (Applied Biosystems; see Table S2 for details of loci and PCR amplification; see also Gossieaux et al., 2018). Allele lengths were determined using GeneMapper V6 (Applied Biosystems).

Parentage assignment was performed using both CERVUS V3.0.7 (Kalinowski et al., 2007) and COLONY V2.0.6.6 (Jones & Wang, 2010). In brief, a first parental pair assignation was performed using likelihood approach with a 90% confidence level in CERVUS. A second separate assignment was also performed with the full‐likelihood method in COLONY. The resulting parental assignations from both software were then compared and when both software suggested the same parent pair for an existing cross (Table S1), the pair was successfully assigned. If that pair was not an existing cross, the next most probable existing pair was then assigned, and so on. If both software did not suggest the same pair, the most probable one between the possible crosses was assigned. The inability to assign both parents to an individual resulted in failed assignations. Unassigned individuals were not included in the analyses.

### 2.6 Statistical analyses

We first used linear mixed models to assess the influence of each parental treatment (temperature and selection) on each body size variable (body mass, length and Fulton's index) at three different periods for their offspring: (1) the first year in laboratory environment, in September 2019 (total *N* = 803), (2) the second year in laboratory environment, in June 2020 (total *N* = 1368) and (3) in September 2020 for second year fish stocked in natural lakes (177 included in the analysis, following parental assignment). For each body size variable at each period, a separate model, including temperature (as a 2‐level factor, i.e. Warm or Cold), selection (as a 2‐level factor, i.e. Selected or Unselected), as well as the interaction between them, was built. To quantify the amount of variance explained by other parental effects (both genetic and environmental), the identity of both parents was also included in the models as random effects and was tested for significance using likelihood ratio tests (LRTs). The model was simplified by a backward elimination procedure, where the least significant term, based on *p*‐value, was sequentially removed until all remaining variables were significant (i.e., *p* ≤ 0.05, confirmed by an LRT). Prior to those analyses, body mass data were taken in September 2020 (i.e. second‐year fish in natural lakes) and were transformed (via a log+1 function) to achieve normality.

For the second year in the lake environment period, a generalized linear mixed model (using logit link and binomial proportion error structure) was used to assess the impact of both treatments and their interaction, as well as the impact of family mean body size on survival. For each family, survival was estimated as the proportion of stocked fish that were captured in September 2020 (response variable: number of fish captured per family weighted by the initial number of juveniles stocked per family) and morphological trait values corresponded to the mean value of that trait for all sampled juveniles of that family. As body mass and length were strongly correlated, two separate models were built for each period: (1) a model including only body mass; (2) a model including body length and Fulton's index. Again, to quantify the amount of variance explained by other parental effects, parent identities were included in both models as random effects and were tested for significance using likelihood ratio tests (LRTs).

All analyses were performed using R software 4.0.5 (R Core Team, 2021). Multicollinearity issues were avoided by only including in the same model variables with variance inflation factors <3, as well as correlation coefficients smaller than 0.5. All mixed model analyses were conducted using lme4 package (Bates et al., 2015). Conditional and marginal *R* ^2^ were obtained via the theoretical method of the rsquared function using the rsq package (Zhang, 2021).

## 3 RESULTS

### 3.1 Parentage assignment and sample size of second year fish in lake environment

We assigned 177 out of 184 fish captured 1 year after stocking to a given family (assignment rate = 96%). The number of fish caught per family was highly variable, ranging from 0 to 23 (mean = 6.6), and so was our proxy for survival percentage per family (range 0–13.8%, mean = 4.7%, Table S1 and Figure S2). The number of fish originating from each breeders' temperature treatment greatly differed, as we caught 166 fish originating from the warm temperature treatment, whereas only 11 fish from the cold temperature treatment were sampled (Table S1). The number of captured fish issued from selected and control groups were similar (Table S1; Unselected treatment *N* = 94; Selected treatment *N* = 83). We also observed a large variation in the number of fish caught per parent. Some individuals had no captured offspring, while one of the males (M7UW – see Table S1 and Figure S2) sired 55 out of the 184 individuals captured.

### 3.2 Correlations among traits

Body mass and length were strongly correlated in all sampling periods (Pearson correlations, *r* = 0.95 for all periods) and thus were not included in the same survival analysis model. However, body length and Fulton's index were included in the same survival analysis model since their correlations were much weaker and only significant in the second year in lake environment (*r* = 0.03: first year in laboratory environment; *r* = 0.02: second year in laboratory environment; *r* = 0.43: second year in lake environment, *p* < 0.001).

### 3.3 Effect of breeders' temperature treatment on morphological traits and survival of offspring

**FIGURE 2:**
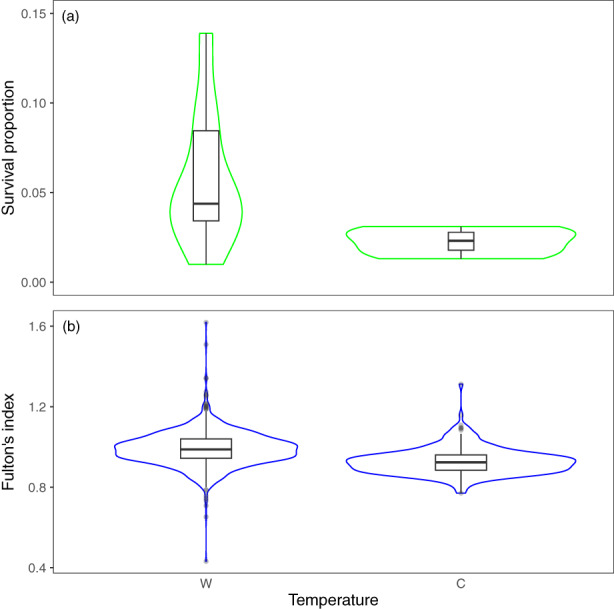
Effect of temperature treatment (C, cold; W, warm) on Brook charr's (a) survival proportion the second year in natural lakes when including body length and Fulton condition index as morphological traits in the model and (b) Fulton condition index the second year in laboratory. Sampling periods are represented by different colours (second year in natural lakes = green, second year in laboratory = blue).

We detected no effect of temperature regime during late stages of gonad maturation of breeders on morphological traits of offspring in the first year in laboratory environment (Tables S3–S5). Similarly, no effect was found on morphological traits of offspring after 1 year in natural lakes, but we found that offspring originating from adults from the cold temperature treatment had lower survival than those from the warm treatment (Table 1 and Figures 2a and S3). Offspring from parents exposed to cold temperature had a smaller Fulton condition index than those from parents exposed to warm temperature on the second year in laboratory environment (−0.056 ± 0.026, *Z* = 2.15, *p* = 0.032, model's marginal *R* ^2^ = 0.065 and conditional *R* ^2^ = 0.304; Figure 2b and Table S5).

**TABLE 1:** Effects of morphological traits on Brook charr survival in natural lakes.

	Estimate	SE	*Z* value	Variance of the random effect	*p*‐Value
Model including only body mass (Marginal *R* ^2^ = 0.031; Conditional *R* ^2^ = 0.081)					
Intercept	−2.949	0.150	19.670		<0.001
Mean body mass of the family (g)	0.002	0.003	0.496		0.62
**Temperature (C)**	**−0.884**	**0.428**	**2.065**		**0.041**
Selection (S)	−0.480	0.255	1.880		0.08
Temperature X selection	0.380	0.818	0.464		0.64
Dam ID (random)				<0.001	1.00
**Sire ID (random)**				**0.180**	**0.001**
Model including body length and Fulton's index (Marginal *R* ^2^ = 0.058; Conditional *R* ^2^ = 0.085)					
Intercept	−6.806	1.384	4.918		<0.001
**Mean Fulton index of the family**	**4.678**	**1.656**	**2.826**		**0.010**
Mean body length of the family (cm)	−0.003	0.039	0.087		0.93
**Temperature (C)**	**−0.763**	**0.382**	**1.996**		**0.043**
Selection (S)	0.038	0.366	0.103		0.92
Temperature X selection	0.341	0.797	0.428		0.67
Dam ID (random)				0.000	1.00
**Sire ID (random)**				**0.097**	**0.004**

*Note*: Final generalized linear mixed models representing the effect of treatments and mean family body mass, length and Fulton's index in a natural environment in September 2020, on Brook charr survival 1 year after stocking. Dam and sire identities were included in both models as random effects. Bold estimates are significant.

### 3.4 Effect of breeders' selection treatment on morphological traits and survival of offspring

**FIGURE 3:**
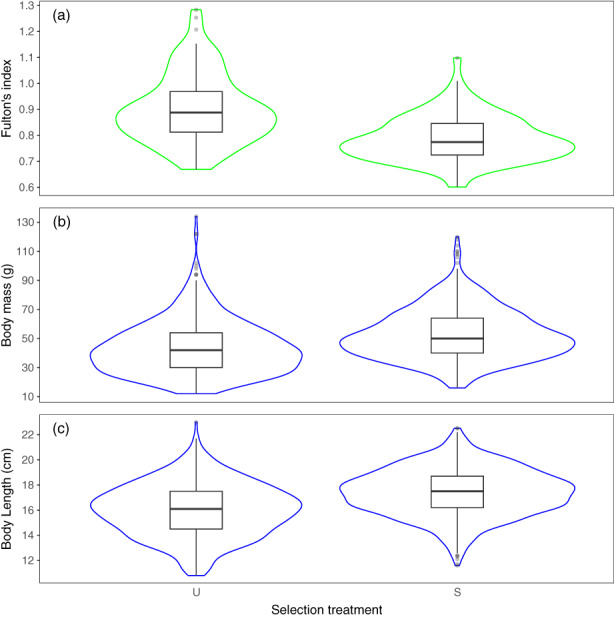
Effect of selection treatment (S, selected; U, unselected) on Brook charr's (a) Fulton condition index the second year in natural lakes, (b) body mass (g) the second year in laboratory environment and (c) body length (cm) the second year in laboratory environment. Sampling periods are represented by different colours (second year in natural lakes = green, second year in laboratory = blue).

We detected no effect of the selection treatment on morphological traits of offspring in the first year in laboratory environment (Tables S3–S5). In fish that spent a year in natural lakes, we found a significant effect of the selection treatment on Fulton's condition index. At this period, fish originating from the selected group had a lower condition index than those from the unselected group (−0.115 ± 0.017, *Z* = 6.72, *p* < 0.001, model *R* ^2^ = 0.205, Figure 3a and Table S5). We found no effect of the selection treatment on survival. However, on the second year in laboratory environment, fish from the selected group were, on average, 21% heavier (effect: 9.024 ± 4.448, *Z* = 2.03, *p* = 0.042, model's marginal *R* ^2^ = 0.056 and conditional *R* ^2^ = 0.250) and 9% longer (effect: 1.407 ± 0.479, *Z* = 2.94, *p* = 0.005, model's marginal *R* ^2^ = 0.105 and conditional *R* ^2^ = 0.278) than unselected fish (Figure 3b,c and Tables S3 and S4).

### 3.5 Effect of parental identity on morphological traits and survival of offspring

We found that body mass, length and Fulton's index were all influenced by the identity of both parents on the first and second year in laboratory environment (Tables S3–S5). On the second year in natural lakes, only dam identity influenced body mass and length, but it did not influence Fulton's index (Tables S3–S5). In the survival analysis of second year fish in natural lakes, sire ID was a significant random effect in both models (model including only mass: LRT: χ^2^ = 11.25, *p* = 0.001, conditional *R* ^2^ = 0.081; model including body length and Fulton condition index: LRT: χ^2^ = 8.14, *p* = 0.004, conditional *R* ^2^ = 0.085).

### 3.6 Effect of offspring morphological traits on survival

We found a positive relationship between Fulton condition index, but not body mass or length, and survival on the second year after stocking in natural lakes (4.678 ± 1.656, *Z* = 2.83, *p* = 0.005, marginal *R* ^2^ = 0.058, see Figure S4).

## 4 DISCUSSION

We found transgenerational effects, resulting from temperature variation during final stages of gonad maturation. The cold temperature treatment was related to different effects on morphological traits, depending on the sampling period. The cold treatment was also related to lower offspring survival the second year in natural lakes. We also found that the selection treatment was related to morphological traits in various ways, depending on the sampling period, but we found no effect of this treatment on survival. The importance of parental identity on morphological traits and survival also greatly differed between sampling periods. Finally, the only relationship we found between morphological traits and survival was the positive effect of Fulton's index on survival for second year fish in natural lakes.

### 4.1 Effect of temperature treatment on morphological traits and survival

In our study, the cold temperature treatment was associated with lower survival on the second year in natural conditions. In the presence of adaptive transgenerational effects, we should expect fish originating from cold acclimated parents to have a higher survival rate than those from warm acclimated parents, as shown by Shama et al. (2014) in a study conducted on marine sticklebacks (*Gasterosteus aculeatus*). The opposite trend observed in our study is thus intriguing. A potential explanation for our results is that lakes where fish were stocked were unusually warm in 2020 and that adaptive transgenerational effects led to an increase in the survival of fish from the warm temperature treatment. This may be possible since the July–August 2020 period was the warmest in the province of Québec in at least 100 years, surpassing 1981–2010 normal by 1.8°C (see information from MLECC at www.environnement.gouv.qc.ca), which nearly corresponds to the temperature difference experienced by warm acclimated compared to cold acclimated parents (+2°C) in our study. Maladaptive transgenerational effects related to cold treatment (ex. due to physiological constraints on egg development, flawed cue perception, etc.) is most likely not an issue in our study considering that colder conditions have been encountered in our production facilities in the past (7°C–9.5°C in mid‐November from 1993 to 2003) without any indications of reproductive failure (e.g. Bastien et al., 2011). Yet, it could also be argued that the difference in the number of families used among temperature treatment (22 warm vs only 5 cold) may have slightly affected the amount of genetic variation present and thus the adaptive potential of fish. Also, we acknowledge that both the low survival observed here as well as the small number of families used in the cold treatment call for caution in the interpretation of our results.

We also found that Fulton's index of second‐year fish in laboratory environment was influenced by the temperature treatment, with fish from cold‐acclimated parents having a lower index than those from warm‐origin adults. This result contrasts with those presented in Penney et al. (2022), where Brook charr juveniles from cold‐acclimated parents had better body condition than those from warm‐acclimated parents. In this study, however, differences in temperature between cold and warm treatment were more important (10°C or 21°C, for cold and warm respectively), and were maintained for a longer period in separate treatments (from May until fall reproduction) than in our study. Further studies under various temperature conditions are needed to conclude the generality of our results.

### 4.2 Effect of selection treatment on morphological traits and survival

The second year in laboratory environment, body mass and length were influenced by the selection treatment experienced by parents, with offspring from selected parents being bigger and longer than the ones from unselected adults, as expected considering the nature of the selection treatment. Interestingly, on the second year in natural lakes, the selection treatment had no impact on body mass or length but led to lower Fulton's index. This could be explained by differences in selective pressures encountered in the wild versus controlled conditions. The latter is usually associated with more relaxed natural selection conditions, mostly due to the absence of predators and parasites, which could allow fish from the selection treatment to achieve their full potential at getting bigger and having faster growth than those from unselected origin. In the wild, selective pressures are usually stronger, possibly mitigating the effect of the selection treatment and limiting the growth of fish from the selected treatment (Evans et al., 2014; Williams & Hoffman, 2009). It may also suggest that unselected fish could outperform selected ones in natural lakes if they carried traits that were beneficial to growth in natural conditions such as feeding behaviour that could be altered by selection treatment (Biro et al., 2004). This result is relevant for Brook charr and other salmonids subjected to planned breeding/stocking programmes, and it underlines the importance of assessing the impact of artificial selection treatments both in laboratory conditions and in natural environments. In our study, the unselected treatment probably includes some domestication effects that might not be present in wild fish. Experiments comparing the behaviour of Laval strain and wild fry, raised in the same facilities, are currently being developed to provide further information about such effects.

### 4.3 Effect of parental identity on morphological traits and survival

Only parental identity explained variation in morphological traits during the first year in laboratory environment. This result confirms the findings of previous studies showing that the development of morphological traits at an early stage is mainly modulated by parental/genetic, rather than environmental, effects in juvenile Brook charr (Crespel et al., 2013; Perry et al., 2004). Parental identity also accounted for an important portion of variation underlying morphological traits in the second year in laboratory environment. However, on the second year in natural lakes, only maternal identity was significant in explaining variation of body mass and length, and no parental effect was detected on Fulton's index. Interestingly, sire identity explained a significant proportion of the variation in survival at that same period. Our findings are in line with those of Penney et al. (2022) that revealed a greater contribution of sires, relative to dams, to transgenerational effects in Brook charr families produced from parents acclimated to cold and warm temperatures and kept in controlled conditions. Again, however, we recognize that low survival and a small number of families in the cold treatment may have hampered our results.

### 4.4 Effect of morphological traits on survival

The only significant relationship we found between Fulton's index and survival was during the second year for fish in natural lakes, where larger condition was positively related to survival. This is not surprising considering that several fish species have size‐dependent fitness (reviewed in Perez & Munch, 2010). However, this result is worth mentioning considering that in our study, as previously stated, the selection treatment (which aimed at producing fish in better condition) led to lower Fulton's index values of second‐year fish in natural lakes. A lower condition for these selected‐origin fish could thus be indirectly detrimental to their survival during that period. This finding is especially important as domesticated and artificially selected Brook charr are largely stocked in North American lakes (Lehnert et al., 2020; MFFP, 2019; White et al., 2018).

## 5 CONCLUSION

Here we showed that temperature experimented by adults affected the survival of offspring, which suggested either some adaptive effect of warm‐acclimated fish or, less likely, some maladaptive transgenerational effects of cold‐acclimated ones. While further studies are necessary to understand the full extent of this result, the response to the warm treatment reported here (e.g. higher survival) suggests that transgenerational effects may enable Brook charr to keep pace, to some extent, with anticipated changes in environmental temperatures. Since thermal conditions during gonadal development could influence post‐stocking fitness, great care should be taken in the monitoring of temperature during pre‐stocking practices. We also found that the selection treatment, which aimed at enhancing fish growth, had little impact on parental effects related to temperature and led to inconsistent effects on morphological traits, depending on the sampling period and environment. Artificial selection should thus be further studied, to better understand its general consequences on fish, as well as its impact on stocking success. Finally, our study also confirms that paternal effects, which are still understudied, can sometimes have a greater influence on traits than maternal effects.

## CONFLICT OF INTEREST STATEMENT

Louis Bernatchez is editor in chief of Evolutionary Applications. All other authors declare no conflicts of interest.

## Supporting information


Appendix S1.

## Data Availability

Data for this study are available at: doi: 10.5061/dryad.wwpzgmsps.
