# Supplementary material for: Transgenerational effects on body size and survival in Brook charr (Salvelinus fontinalis)
Source: Evol Appl. 2023 May 2;16(5):1061–70. doi: 10.1111/eva.13553 (PMC10197224; doi:10.1111/eva.13553)
Supplement: Supplementary file 1 — Appendix S1. [file EVA-16-1061-s001.docx]

**Supplementary material for:**

**Transgenerational parental and environmental effects on body size and survival in brook charr (*Salvelinus fontinalis*)**

**Table S1.** Number of fish stocked and sampled in natural lakes as well as survival percentage the second year in natural lake environment, for each of the 27 families with individuals surviving until stocking stage.

| Families | Nb stocked | Nb sampled | Survival % |
| --- | --- | --- | --- |
| ♀15UW x ♂2UW | 146 | 5 | 3.42 |
| ♀15UW x ♂7UW | 166 | 23 | 13.86 |
| ♀16UW x ♂11UW | 147 | 8 | 5.44 |
| ♀16UW x ♂9UW | 105 | 9 | 8.57 |
| ♀17UW x ♂11UW | 124 | 0 | 0 |
| ♀18UW x ♂7UW | 135 | 12 | 8.89 |
| ♀18UW x ♂8UW | 118 | 6 | 5.08 |
| ♀19UW x ♂2UW | 140 | 6 | 4.29 |
| ♀19UW x ♂7UW | 144 | 20 | 13.89 |
| ♀1SW x ♂1SW | 163 | 3 | 1.84 |
| ♀1SW x ♂2SW | 169 | 9 | 5.33 |
| ♀1SC x ♂1SC | 153 | 2 | 1.31 |
| ♀2UC x ♂1UC | 103 | 2 | 1.94 |
| ♀2UC x ♂6UC | 112 | 3 | 2.68 |
| ♀2SC x ♂1SC | 129 | 4 | 3.10 |
| ♀2SC x ♂2SC | 83 | 0 | 0 |
| ♀3SW x ♂3SW | 162 | 6 | 3.70 |
| ♀3SW x ♂4SW | 133 | 5 | 3.76 |
| ♀4SW x ♂3SW | 134 | 2 | 1.49 |
| ♀4SW x ♂4SW | 125 | 6 | 4.80 |
| ♀5SW x ♂5SW | 147 | 5 | 3.40 |
| ♀5SW x ♂6SW | 127 | 13 | 10.24 |
| ♀6SW x ♂5SW | 131 | 4 | 3.05 |
| ♀6SW x ♂6SW | 137 | 5 | 3.65 |
| ♀7SW x ♂7SW | 137 | 6 | 4.38 |
| ♀7SW x ♂8SW | 142 | 12 | 8.45 |
| ♀8SW x ♂7SW | 101 | 1 | 0.99 |

**Table S2.** Characteristics of microsatellite loci used to genotype *Salvelinus fontinalis* with their names, repeat motifs, allele size range, MgCl_2_ concentration (mM) used in PCRs, annealing temperature, number of cycles used for PCR amplification and reference.

| **Locus** | **Repeat motif** | **Range**  **(bp)** | **MgCl_2_**  **(mM)** | **Annealing**  **temperature**  **(^o^C)** | **Number of cycles** | **Reference** |
| --- | --- | --- | --- | --- | --- | --- |
| *Sco218* | (GATA)_31_ | 130–214 | 1 | 56 | 35 | Dehaan & Ardren, 2005 |
| *Sfo*B52 | (GCGT)_12_ | 189–229 | 1.2 | 60 | 35 | King *et al.*, 2012 |
| *Sfo*C24 | (GAT)_10_ | 103–124 | 1 | 58 | 30 | King *et al.*, 2012 |
| *Sfo*C86 | (GAT)_8_ | 91–124 | 1.5 | 58 | 30 | King *et al.*, 2012 |
| *Sfo*C113 | (GAT)_12_ | 125–155 | 1.2 | 56 | 35 | King *et al.*, 2012 |
| *Sfo*C88 | (GAT)_16_ | 167–194 | 1 | 54 | 35 | King *et al.*, 2012 |
| *Sfo*D75 | (TAGA)_17_ | 160–228 | 1 | 58 | 35 | King *et al.*, 2012 |
| *Sfo*D100 | (TAGA)_11_ | 197–245 | 1.2 | 58 | 35 | King *et al.*, 2012 |
| *Sfo*C115 | (CTCA)_21_ | 218–347 | 1.2 | 60 | 35 | King *et al.*, 2012 |
| *Ssa85* | (GT)_14_ | 95–133 | 1 | 60 | 35 | O’Reilly *et al.*, 1996 |
| *Ssa197* | (GT)_5_C(TG)_4_TC(TG)_3_A(GTGA)_15_ | 138–158 | 1 | 62 | 35 | O’Reilly *et al.*, 1996 |
| *Sfo226Lav* | (TG)_21_(CGTG)_13_ | 335–389 | 1 | 60 | 35 | Perry *et al.*, 2005 |

**Table S3.** Final linear mixed model representing the effect of treatment on brook charr body mass at three different period: 1) the first year in laboratory environment in September 2019, 2) in June 2020, second year in laboratory environment, and 3) the second year in natural lake environment, in September 2020. Dam and sire identities were included in all models as random effects. Bold estimates are significant.

|  | Estimate | SE | Z value | Variance of random effect | P-value |
| --- | --- | --- | --- | --- | --- |
| First year in laboratory environment (Marginal R^2^ = 0.000; Conditional R^2^ = 0.292) | | | | | |
| Intercept | 7.369 | 0.445 | 16.580 |  | <0.001 |
| Selection(S) | 0.219 | 0.884 | 0.248 |  | 0.79 |
| Temperature(C) | -1.795 | 1.026 | 1.750 |  | 0.08 |
| Selection X Temperature | 2.723 | 2.094 | 1.300 |  | 0.16 |
| **Dam ID (random)** |  |  |  | **0.999** | **<0.001** |
| **Sire ID (random)** |  |  |  | **1.981** | **<0.001** |
| Second year in laboratory environment (Marginal R^2^ = 0.056; Conditional R^2^ = 0.250) | | | | | |
| Intercept | 43.238 | 3.502 | 12.347 |  | <0.001 |
| **Selection(S)** | **9.024** | **4.448** | **2.029** |  | **0.042** |
| Temperature(C) | -7.052 | 5.062 | 1.393 |  | 0.14 |
| Selection X Temperature | 3.682 | 10.567 | 0.348 |  | 0.71 |
| **Dam ID (random)** |  |  |  | **28.230** | **<0.001** |
| **Sire ID (random)** |  |  |  | **39.170** | **<0.001** |
| Second year in natural lake environment* (Marginal R^2^ = 0.000; Conditional R^2^ = 0.310) | | | | | |
| Intercept | 3.979 | 0.124 | 32.210 |  | <0.001 |
| Selection(S) | -0.198 | 0.255 | 0.777 |  | 0.34 |
| Temperature(C) | -0.348 | 0.333 | 1.044 |  | 0.28 |
| Selection X Temperature | 0.982 | 0.631 | 1.556 |  | 0.08 |
| **Dam ID (random)** |  |  |  | **0.165** | **0.001** |
| Sire ID (random) |  |  |  | <0.001 | 1.00 |

*For this period, body mass was transformed via a (log+1) transformation to achieve normality.

**Table S4.** Final linear mixed model representing the effect of treatment on brook charr body length at three different period: 1) the first year in laboratory environment in September 2019, 2) in June 2020, second year in laboratory environment, and 3) the second year in natural lake environment, in September 2020. Dam and sire identities were included in all models as random effects. Bold estimates are significant.

|  | Estimate | SE | Z value | Variance of random effect | P-value |
| --- | --- | --- | --- | --- | --- |
| First year in laboratory environment (Marginal R^2^ = 0.000; Conditional R^2^ = 0.335) | | | | | |
| Intercept | 9.409 | 0.180 | 52.380 |  | <0.001 |
| Selection(S) | 0.452 | 0.343 | 1.317 |  | 0.16 |
| Temperature(C) | -0.707 | 0.410 | 1.723 |  | 0.08 |
| Selection X Temperature | 0.896 | 0.808 | 1.109 |  | 0.23 |
| **Dam ID (random)** |  |  |  | **0.109** | **0.003** |
| **Sire ID (random)** |  |  |  | **0.386** | **<0.001** |
| Second year in laboratory environment (Marginal R^2^ = 0.105; Conditional R^2^ = 0.278) | | | | | |
| Intercept | 15.975 | 0.375 | 42.580 |  | <0.001 |
| **Selection(S)** | **1.407** | **0.479** | 2.940 |  | **0.005** |
| Temperature(C) | -0.512 | 0.571 | 0.897 |  | 0.33 |
| Selection X Temperature | 0.419 | 1.207 | 0.347 |  | 0.70 |
| **Dam ID (random)** |  |  |  | **0.488** | **<0.001** |
| **Sire ID (random)** |  |  |  | **0.287** | **<0.001** |
| Second year in natural lake environment (Marginal R^2^ = 0.000; Conditional R^2^ = 0.320) | | | |  |  |
| Intercept | 19.021 | 0.724 | 26.290 |  | <0.001 |
| Selection(S) | -0.340 | 1.603 | 0.212 |  | 0.73 |
| Temperature(C) | -1.604 | 1.974 | 0.812 |  | 0.40 |
| Selection X Temperature | 5.677 | 3.931 | 1.444 |  | 0.09 |
| **Dam ID (random)** |  |  |  | **5.695** | **<0.001** |
| Sire ID (random) |  |  |  | <0.001 | 1.00 |

**Table S5**. Final linear mixed model representing the effect of treatment on brook charr Fulton’s body condition index at three different period: 1) the first year in laboratory environment in September 2019, 2) in June 2020, second year in laboratory environment, and 3) the second year in natural lake environment, in September 2020. Dam and sire identities were included in all models as random effects. Bold estimates are significant.

|  | Estimate | SE | Z value | Variance of random effect | P-value |
| --- | --- | --- | --- | --- | --- |
| First year in laboratory environment (Marginal R^2^ = 0.000; Conditional R^2^ = 0.367) | | | | | |
| Intercept | 0.838 | 0.013 | 63.100 |  | <0.001 |
| Selection(S) | -0.043 | 0.026 | 1.661 |  | 0.09 |
| Temperature(C) | -0.044 | 0.030 | 1.458 |  | 0.13 |
| Selection X Temperature | 0.049 | 0.062 | 0.788 |  | 0.39 |
| **Dam ID (random)** |  |  |  | **0.001** | **<0.001** |
| **Sire ID (random)** |  |  |  | **0.001** | **<0.001** |
| Second year in laboratory environment (Marginal R^2^ = 0.065; Conditional R^2^ = 0.304) | | | | | |
| Intercept | 0.989 | 0.012 | 80.024 |  | <0.001 |
| Selection(S) | -0.039 | 0.021 | 1.81 |  | 0.06 |
| **Temperature(C)** | **-0.056** | **0.026** | **2.154** |  | **0.032** |
| Selection X Temperature | 0.025 | 0.051 | 0.486 |  | 0.60 |
| **Dam ID (random)** |  |  |  | **0.001** | **<0.001** |
| **Sire ID (random)** |  |  |  | **0.001** | **<0.001** |
| Second year in natural lake environment (Marginal R^2^ = 0.205; Conditional R^2^ = 0.000) | | | | | |
| Intercept | 0.902 | 0.012 | 77.126 |  | <0.001 |
| **Selection(S)** | **-0.115** | **0.017** | **6.717** |  | **<0.001** |
| Temperature(C) | -0.040 | 0.035 | 1.128 |  | 0.26 |
| Selection X Temperature | 0.059 | 0.071 | 0.829 |  | 0.41 |
| Dam ID (random) |  |  |  | <0.001 | 1.00 |
| Sire ID (random) |  |  |  | <0.001 | 1.00 |


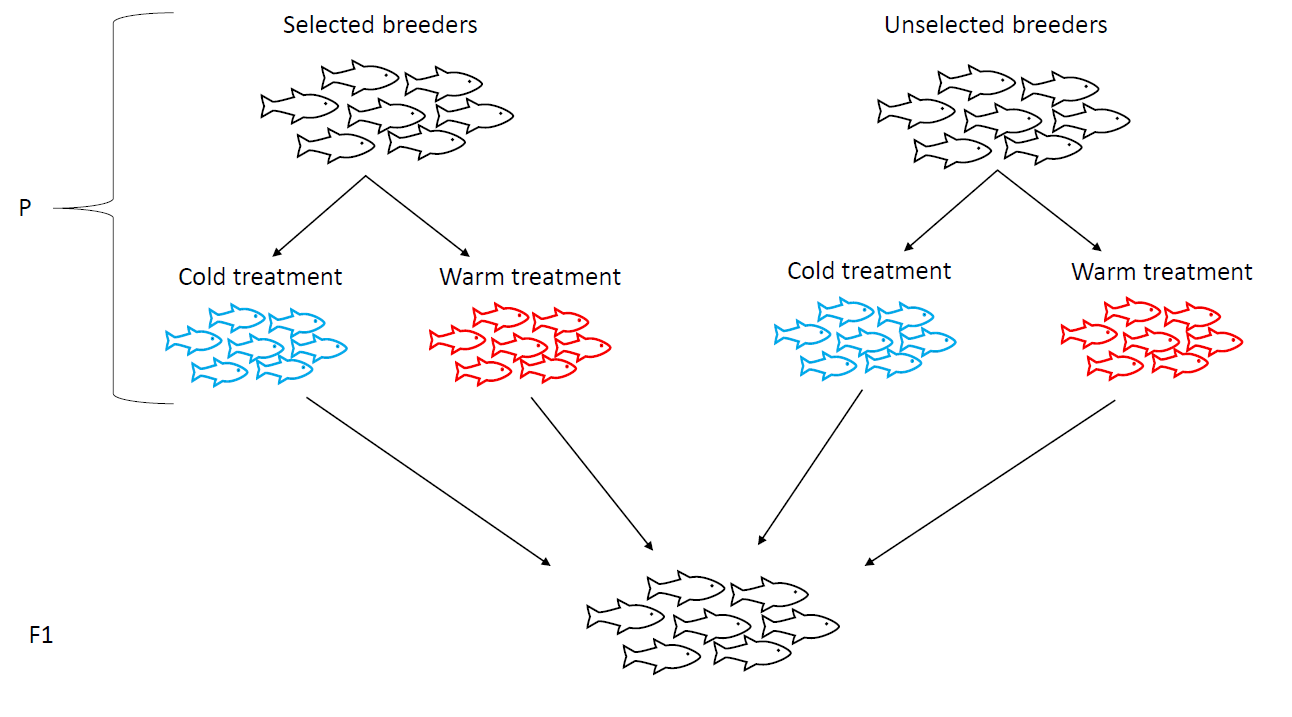


**Figure S1.** Schematic illustration of the rearing methods. Two parental lines of breeders (P) were present in this study 1) a line of selected breeders (selection program aiming to eliminate sexual maturation and improve growth at age 1) and 2) a line of unselected breeders, on which no selection process was applied (even though a domestication process certainly occurred). Breeders from both selection regimes were separated into two groups in order to expose them, during the late gonad maturation period, to two different thermal regimes: a "Cold" group with temperature gradually decreasing from 11.5°C in September to 3°C in December, and a "Warm" group with temperature gradually decreasing from 13.5°C in September to 5°C in December).

**Figure S2.** Number of fish caught per family the second year in natural lake environment (September 2020). Parental identities are followed by absolute number of fish caught from that family, and the overall percentage of captures that it represents. Parents ID are composed of the following letters: F for female or M for male; S for selected or U for unselected and C for cold or W for warm.


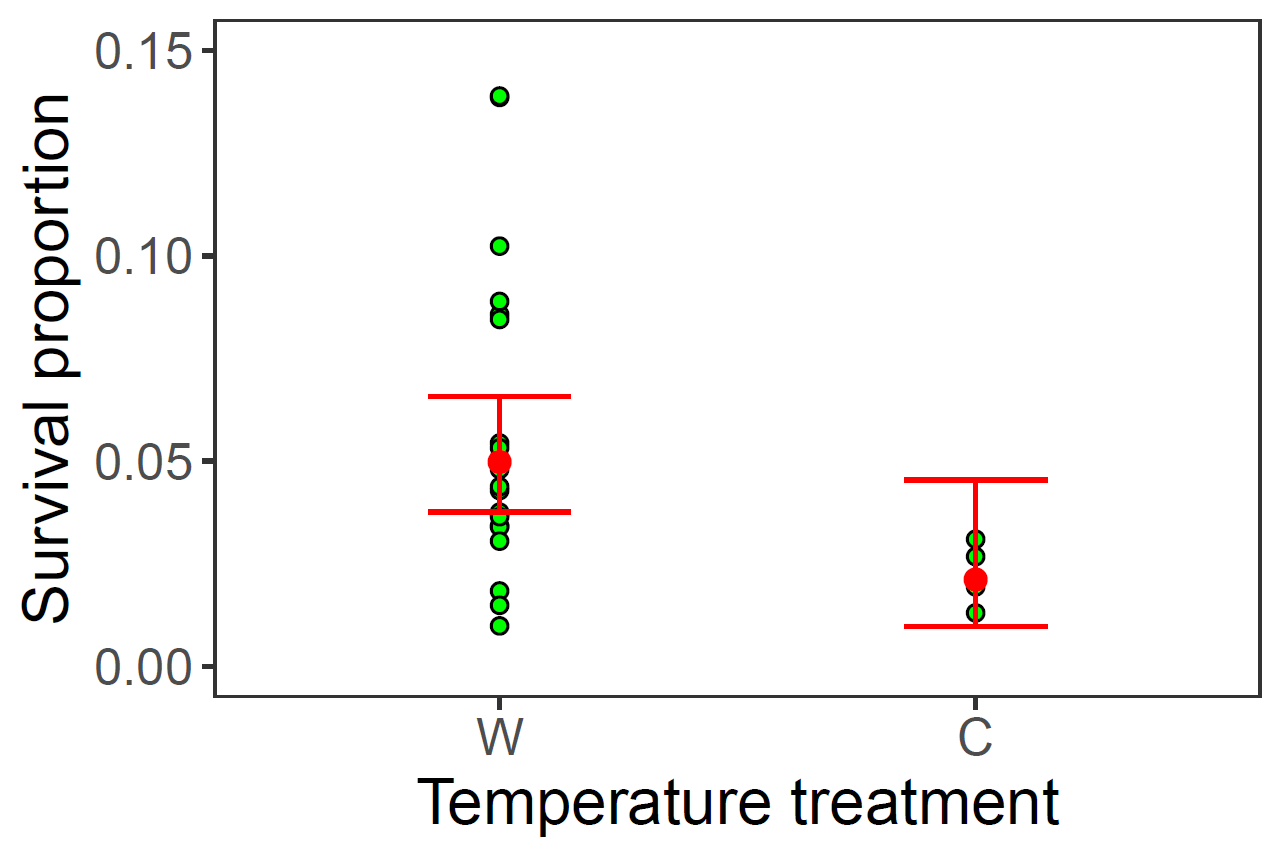


**Figure S3.** Effect of temperature treatment (W=Warm, C=Cold) on survival proportion the second year in natural lake environment, when including only body mass as a morphological trait in the model.

**
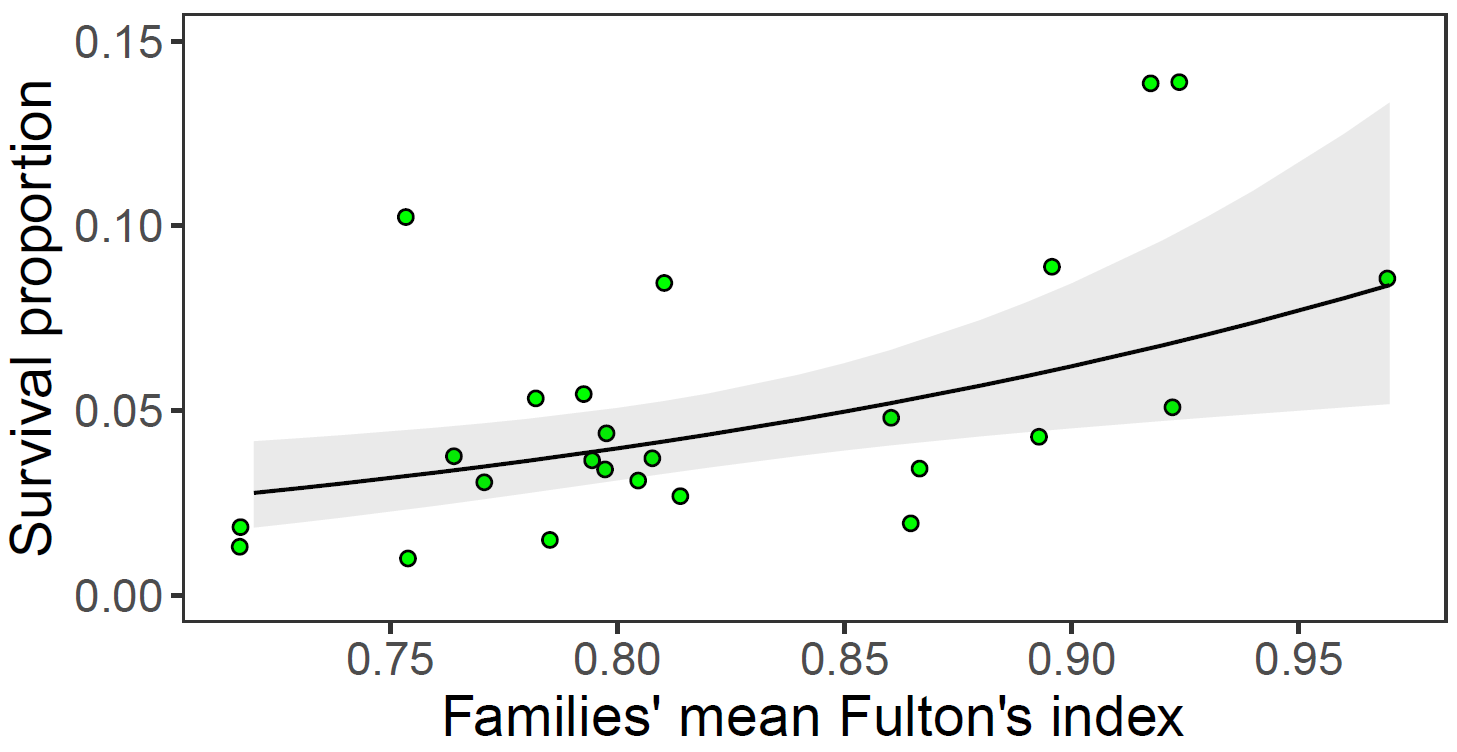
Figure S4.** Brook charr’s survival proportion the second year in natural lakes in relation to families’ mean Fulton condition index in that period. 95% confidence intervals are represented in grey.
